# Phosphate signaling through alternate conformations of the PstSCAB phosphate transporter

**DOI:** 10.1186/s12866-017-1126-z

**Published:** 2018-01-19

**Authors:** Ramesh K. Vuppada, Colby R. Hansen, Kirsta A. P. Strickland, Keilen M. Kelly, William R. McCleary

**Affiliations:** Department of Microbiology and Molecular Biology, Brigham Young University, Provo, UT USA

**Keywords:** Phosphate homeostasis, Two-component signal transduction, Histidine kinase, ABC transporter

## Abstract

**Background:**

Phosphate is an essential compound for life. *Escherichia coli* employs a signal transduction pathway that controls the expression of genes that are required for the high-affinity acquisition of phosphate and the utilization of alternate sources of phosphorous. These genes are only expressed when environmental phosphate is limiting. The seven genes for this signaling pathway encode the two-component regulatory proteins PhoB and PhoR, as well as the high-affinity phosphate transporter PstSCAB and an auxiliary protein called PhoU. As the sensor kinase PhoR has no periplasmic sensory domain, the mechanism by which these cells sense environmental phosphate is not known. This paper explores the hypothesis that it is the alternating conformations of the PstSCAB transporter which are formed as part of the normal phosphate transport cycle that signal phosphate sufficiency or phosphate limitation.

**Results:**

We tested two variants of PstB that are predicted to lock the protein in either of two conformations for their signaling output. We observed that the *pstB*Q160K mutant, predicted to reside in an inward-facing, open conformation signaled phosphate sufficiency whereas the *pstB*E179Q mutant, predicted to reside in an outward-facing, closed conformation signaled phosphate starvation. Neither mutant showed phosphate transport.

**Conclusions:**

These results support the hypothesis that the alternating conformations of the PstSCAB transporter are sensed by PhoR and PhoU. This sensory mechanism thus controls the alternate autokinase and phospho-PhoB phosphatase activities of PhoR, which ultimately control the signaling state of the response regulator PhoB.

## Background

Inorganic phosphate (Pi) is an essential compound for a cell’s energy metabolism and is a component of nucleic acids, phospholipids and other cell constituents. Bacterial cells must maintain intracellular Pi pools for optimal growth and they have developed intricate strategies to sense Pi and control the expression of genes to best fit their environmental circumstances. The basic principles underlying how these simple cells alter their gene expression based upon the availability of environmental Pi are generally known [1–4]. However, some of the molecular mechanisms by which cells sense Pi and how they process that information to alter a cell’s transcription machinery are not yet fully understood.

**Fig. 1:**
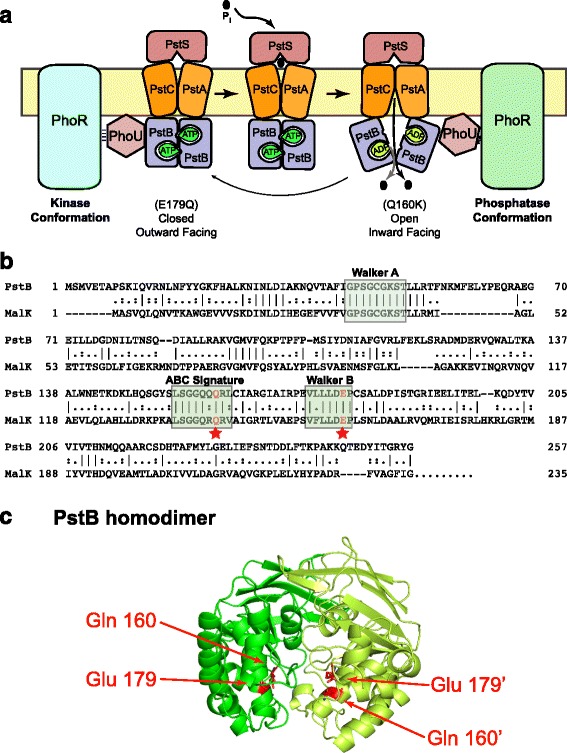
Conformational signaling model for control of the Pho regulon. **a** As the Pst transporter alternates between its inward and outward-facing conformations during Pi transport, it directly interacts with PhoR and PhoU. The inward facing conformation, which is stabilized by the *pstB*Q160K mutation, interacts with PhoR to stabilize its phosphatase conformation (light blue) while the outward conformation, which is stabilized by the *pstB*E179Q mutation, favors the kinase conformation of PhoR (green). **b** Sequence alignment of PstB and MalK performed by the European Molecular Biology Open Software Suite (EMBOSS). The mutated residues are marked with red stars. The Walker A, Walker B and the ABC Signature motifs are highlighted by green boxes. Amino acid identities are shows by vertical lines and conserved residues are shown by dots. **c** A three-dimensional model of the PstB homodimer created using the SWISS-MODEL website. The Q160 and E179 amino acid residues of PstB are highlighted. The Q160 and E179 residues are located on the dimer interface and are normally involved in ATP binding and hydrolysis

The genes under control of the Pi regulatory system are called the Pho regulon. These genes are positively regulated in response to limiting external Pi levels and include *phoA,* the gene for the periplasmic enzyme alkaline phosphatase (AP) that is often used as a reporter of the signaling status of the regulon [5, 6]. The PhoBR two-component system plays a central role in controlling the Pho regulon (See Fig. 1a) [7, 8]. PhoR is the sensor histidine kinase/phospho-PhoB phosphatase [8–10]. It consists of an N-terminal membrane domain, a PAS domain, a DHp domain, and a C-terminal CA domain. PAS domains function in signal perception in a wide variety of organisms with its name being derived from the *Drosophila* proteins **P**er, **A**RNT and **S**im [11, 12]. Under Pi-limiting conditions PhoR autophosphorylates on a conserved histidine residue located in the DHp domain (Dimerization and Histidine Phosphorylation [13]) and serves as the phospho-donor to PhoB. The CA domain of PhoR derives its name by having **c**atalytic and **A**TP-binding functions [13]. In Pi-rich environments PhoR dephosphorylates phospho-PhoB by employing its phosphatase activity [10]. Since PhoR does not have a significant periplasmic domain that would bind to Pi, it remains unclear how PhoR perceives external Pi in order to regulate its opposing autokinase and phospho-PhoB phosphatase activities. PhoB, the response regulator of this two-component system, binds to conserved DNA sequences that are located upstream of regulated genes when it is phosphorylated and then interacts with RNA polymerase to activate transcription [14–19].

In addition to PhoR and PhoB, the phosphate-specific transporter PstSCAB and the PhoU proteins are required for Pi signal transduction [20]. It has been proposed that the PstSCAB transporter is the ultimate sensor of external Pi [2], but how it communicates this information about Pi levels is unknown. In high-Pi growth environments the PstSCAB transporter and PhoU negatively regulate the kinase activity of PhoR and stimulate its phospho-PhoB phosphatase activity. Deletion mutations of any one of the transporter genes or *phoU* prevent signal transduction and lead to overexpression of the Pho regulon, which shows that the default state of the signaling pathway is in a “kinase-on” mode [1]. Pi signaling therefore involves the activation of the phosphatase function of PhoR. The autokinase activity of PhoR is stimulated and its phospho-PhoB phosphatase activity is inhibited in low-Pi environments. Each of the seven signal transduction genes is part of the Pho regulon, which creates a positive feedback loop to amplify the Pi starvation signal [1, 2].

PstSCAB is a member of the ATP-binding cassette (ABC) transporter superfamily [21–23]. These transporters are widespread in nature and can serve as importers or exporters [24–26]. PstSCAB belongs to a class of importers that consists of an extracellular ligand binding protein, a transmembrane domain (TMD) that forms the channel through which the substrate will pass and a dimeric nucleotide-binding domain (NBD) where ATP is bound and hydrolyzed [27, 28]. In the Pst transporter, PstS is the periplasmic phosphate binding protein that presents Pi to the TMD. PstC and PstA make up the TMD and PstB is the dimeric NBD that powers transport [1]. The NBD includes several highly-conserved motifs that are required for function. For example, the Walker A motif, the Walker B motif and the ABC signature motif are important regions of the protein that are responsible for ATP binding, ATP hydrolysis and stabilizing the bound ATP, respectively [24, 25, 29]. ATP binding and hydrolysis power the transport of substances across the membrane through an alternating access mechanism in which a substrate is first bound to an outward-facing TMD that is formed as ATP is bound across a closed NBD dimer. This binding event triggers ATP hydrolysis, which leads to the opening of the NBD dimer and the associated adoption of an inward-facing conformation of the TMD that releases the substrate into the cytoplasm (See Fig. 1a).

Recent studies have shown that PhoU directly interacts with PstB and PhoR, suggesting a signaling mechanism in which PhoU binds to the PstB component of the PstSCAB transporter and relays environmental Pi levels to PhoR, which then modulates its kinase and phosphatase activities [30, 31]. One possibility of how the Pst transporter controls the partitioning of the kinase and phosphatase activities of PhoR could be by controlling the intracellular Pi concentration, which could be sensed by the cytoplasmic PAS domain of PhoR. However, ^31^P nuclear magnetic resonance studies have shown that intracellular Pi levels remain constant irrespective of the signaling status of the Pho regulon [32], suggesting that the intracellular Pi level is not responsible for the signal to PhoR from the Pst transporter. Another possibility that has been suggested is that PhoR senses the transport activity of PstSCAB [33]. Countering this proposal are genetic studies of PstC and PstA that separated the transport activity of the protein from its signaling activity [34, 35]. When Arg220 of PstA and Arg237 and Glu240 of PstC were mutated to glutamine residues, Pi transport was blocked but Pi signaling still occurred [34, 35].

Considerable work on the homologous maltose transporter has suggested a refinement to the activity-sensing hypothesis to explain Pi signaling. The maltose ABC transporter from *E. coli* is a model to study the structure and mechanisms of ABC transporters [24, 36–38]. Its periplasmic binding protein is MalE, its TMD is made of MalF and MalG, and its NBD is MalK. Studies have shown that the conformational changes triggered in the NBD domain by binding and hydrolyzing ATP are essential for substrate transport across the membrane. Daus et al. demonstrated that a MalK Q140K mutant, which contains a mutation in the ABC signature motif, and MalK E159Q, which contains a mutation in the Walker B motif, are locked in the open and closed conformations, respectively [37]. They used cross-linkers of different lengths to probe the conformational states of the MalK dimer in these mutants. Moreover, the crystal structure of the entire maltose transporter with the E159Q mutation was solved by Oldham et al. showing that it crystallized in the closed conformation [36].

Based upon these studies, we hypothesize that the two signaling states of the PstSCAB transporter correspond to the two conformational states of the transport cycle; an outward facing, PstB-closed form primed for Pi import and an inward facing, PstB-open conformation not primed for Pi import (See Fig. 1a). In the outward conformation, ATP would be tightly bound across the PstB dimer interface. We assume that this is the ground state of the transporter. This form would be present when Pi levels are low and it would promote the “kinase-on/phosphatase-off” state of PhoR. Alternatively, the inward-facing conformation of PstB would be formed following ATP hydrolysis when Pi is present and is being actively transported. We posit that this conformation of the transporter promotes the “phosphatase-on/kinase-off” form of PhoR. When Pi-bound PstS interacts with the PstC/PstA transmembrane proteins, it triggers ATP hydrolysis within the PstB dimer and causes the conformational change that releases Pi into the cytosol. Our hypothesis is that the complex of PhoU and PhoR recognizes the alternate conformations of the PstSCAB transporter to modulate PhoR’s alternate activities. In this paper, we test this model by creating *pstB* mutations that are in the same positions as the Q140K and E150Q *malK* mutations described above that are predicted to favor stable inward-facing and outward-facing conformations of the transporter and then assay the signaling states of the system.

## Methods

### Strains, plasmids and media

*E. coli* BW25113 was used as the wild-type strain [39]. Strain BW26337 contains a Δ*pstSCAB-phoU*::FRT mutation and BW26388 harbors a Δ*pstB*::kan mutation [39]. The parent plasmid used in this study is pRR48 [40]. It is a medium copy number plasmid that confers ampicillin resistance and expresses cloned genes from a *Tac* promoter. p48SCABU plasmid was constructed by amplifying the *pstSCAB-phoU* operon by PCR using primers that contained embedded *Nde*I and *Kpn*I restriction sites. The PCR product was digested with those enzymes and ligated into a similarly digested pRR48 plasmid vector. The plasmids encoding mutant versions of *pstB* were p48SCAB(Q160K)U and p48SCAB(E179Q)U. They are derived from p48SCABU and were constructed using the Quik-Change site-directed mutagenesis kit from Agilent Technologies and then their sequences were verified by DNA sequence analysis. p48pstB was constructed in a similar manner by amplifying only the *pstB* gene. p48pstBHis encodes the PstB protein with a 6X–His tag at its C-terminus and was constructed as above by incorporating six histidine codons in the appropriate PCR primer. The mutant derivatives of p48pstBHis were constructed by amplifying and cloning the mutant genes from other plasmids. Strains were grown at 37 °C in MOPS (morpholinepropanesulfonic acid) defined minimal medium [41] with either 0.06% glucose and 2.0 mM Pi (MOPS HiPi) or with 0.4% glucose and 0.1 mM Pi (MOPS LoPi) unless otherwise noted. Ampicillin was included at 50 μg/ml.

### Alkaline phosphatase assays

Cultures were grown overnight in MOPS LoPi medium at 37 °C with shaking. 20 μl of overnight cultures were used to inoculate 2 ml MOPS HiPi or MOPS LoPi medium and allowed to grow on a roller drum for 7 h at 37 °C. 600 μl of these cultures were pelleted and then re-suspended in 600 μl of 1 M Tris-HCl pH 8.2. 100 μl of the cell suspension were then added to 100 μl of 1 M Tris-HCl pH 8.2 for a 1:2 dilution and the OD_600_ values were determined with a Thermo Scientific Multiscan FC 96 well plate reader. To the remaining 500 μl of cells, 10 μl of 0.1% sodium dodecyl sulfate and 20 μl of chloroform were added and the tubes were vortexed twice for 5 s at 20 s intervals. 50 μl of each sample were loaded into a 96 well plate containing 150 μl of 1 M Tris-HCl pH 8.2 and incubated at 37 °C for 10 min to equilibrate the temperature. Following the incubation, 40 μl of 20 mM p-nitrophenyl Pi in 1 M Tris-HCl pH 8.2 were added and OD_420_ values were determined at 1 min intervals for 20 min and the maximum kinetic rates for each sample were measured (∆A420/min). Finally, arbitrary AP units were calculated as (1000 × maximum kinetic rate)/(2 × OD_600_ of the overnight culture). Each strain was assayed using two biological replicates in duplicate. The average AP values of each sample with error bars representing standard deviations were reported.

### Measurement of pi depletion and pi-signaling during a growth curve

Cells were grown overnight in MOPS HiPi medium at 37 °C with shaking, pelleted and re-suspended in MOPS minimal medium with 0.06% glucose without Pi. The resuspended cells were then inoculated into flasks containing 40 ml of MOPS minimal medium containing 60 μM Pi and 0.4% glucose to a starting OD_600_ of 0.02 and grown at 37 °C with shaking. 2 ml of cells were collected at hourly intervals of which 1 ml was used to measure the OD_600_ and the other 1 ml of cells were pelleted by centrifugation. The supernatant and pellets were separated and stored at −20 °C for AP and Pi assays. The supernatant’s Pi concentrations were quantified using the commercially available Malachite Green Phosphate Assay Kit by BioAssay Systems as directed by the manufacturer.

### Phosphate uptake measurements

Cells were grown overnight in 5 ml MOPS LoPi containing 0.1 mM IPTG after which they were washed twice with 5 ml MOPS medium free of glucose and Pi. To completely starve the cells of Pi, they were then re-suspended to an OD_600_ of ~0.45 in MOPS medium containing 0.4% glucose and 0.1 mM IPTG, but no Pi. They were then incubated at 37°C on a roller drum for 2 h. Transport assays were performed at room temperature in which 750 μl of cells at an OD_600_ of 0.45 were added to 750 μl of a 10.5 μM solution of K_2_HPO_4_. After 45 s of incubation, 1000 μl of cells were removed and rapidly filtered through pre-wet 0.2 μm nitrocellulose filters using a Millipore 1125 vacuum filter apparatus. The filtrates were collected in glass tubes. Pi concentrations of each filtrate sample were then measured using the Malachite Green assay described above. Pi uptake was determined by subtracting the amount of Pi (in nmoles) in the filtrate from the amount in a blank reaction containing no cells and dividing by the product of the dry weight of cells in each sample and the time. The dry weight of cells was estimated from the following conversion factors: OD_600_ of 1 = 1.11 X 10^9^ cells/ml [42], and each cell has a dry weight of 2.8 X 10^−13^ g. Each strain was assayed using two biological replicates in duplicate.

### Western blots

The immunoblot assays were performed as described previously using a mouse ɑ-Penta-His antibody (Qiagen) [43, 44]. Immunoblots were visualized using the WesternBreeze chemiluminescent Western blot immunodetection kit (Invitrogen).

### Modeling of PstB structure

The PstB sequence was structurally modeled to the *Thermococcus litoralis* MalK crystal structure (Protein Data Bank [45] accession number 1 g29 by using the Swiss-Model automated mode and requesting a homo-dimer structure [46]. The final image was created by using MacPyMol (http://pymol.org/).

## Results

**Fig. 2:**
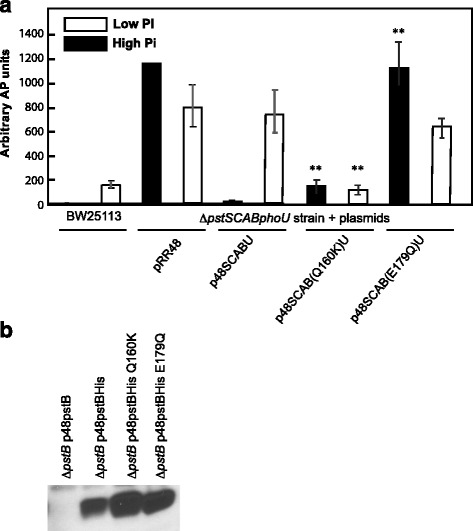
Pi-signaling in wild type cells and experimental strains. **a** AP activity levels for BW25113 (wt) and BW26337 (Δ*pstSCABphoU*) with pRR48, p48SCABU, p48SCAB(Q160K)U or p48SCAB(E179Q)U plasmids. Cells were grown overnight in either MOPS LoPi or MOPS HiPi media, diluted in the morning and grown for an additional seven hours in the indicated medium. Bacterial AP activities were calculated from the averages of three biological replicates performed in duplicate. Error bars represent ± standard error of the mean. **, *P* < 0.01 for the null hypothesis that the means are the same for the AP units between the strain harboring the p48SCABU plasmid grown in either low or high Pi medium and the mutant strains grown in the same medium, as determined by a two-tailed Student t test. **b** Western blot to confirm protein stability. The indicated plasmids were introduced into BW26388, a strain that contains a Δ*pstB*::kan deletion mutation, and the strains were grown overnight in MOPS HiPi medium containing 50 μM IPTG and 50 μg/ml ampicillin and processed for Western blotting

To test the conformational signaling hypothesis we wanted to introduce mutations into *pstB* that are in the same positions as the *malK* mutations that lock that protein into alternate conformations. Figure 1b presents the amino acid alignment of MalK and PstB showing that Q160 and E179 of PstB correspond to Q140 and E159 of MalK. Figure 1c presents a three-dimensional model of the PstB dimeric structure, created using the SWISS-MODEL website [46], which shows the positions of Q160 and E179. As can be seen in this model, these residues lie at the dimer interface of PstB. The Q160K mutation in PstB is predicted to prevent or reduce ATP binding and the E179Q mutation is predicted to prevent ATP hydrolysis [37, 47]. The entire *pstSCAB-phoU* operon was cloned into the medium-copy number plasmid pRR48, which was then tested for complementation of a Δ*pstSCABphoU* chromosomal mutation. As shown in Fig. 2a, the deletion strain (BW26337) containing the empty vector showed high levels of AP activity, regardless of the Pi content of the medium; whereas the strain containing p48pstSCABU complemented the chromosomal deletion by producing high levels of AP when grown in a low-Pi medium and low AP levels in a high-Pi medium. This pattern of gene expression mirrored the wild-type strain, BW25113; although the magnitude of induction was different in the complemented strain, probably because of copy number effects from the plasmid vector. We then tested the Q160K and E179Q *pstB* mutants by introducing the plasmids expressing these genes into the Δ*pstSCABphoU* strain and assaying for AP production following seven hours of growth (Fig. 2a). Our results showed that the *pstB*E179 mutation led to high AP production regardless of the Pi content of the medium and that the *pstB*Q160K mutation displayed lower AP levels under both conditions. The results for the E179Q mutant are similar to the Δ*pstSCABphoU* deletion strain containing an empty vector (Fig. 2a). To confirm that the Q160K and E179Q mutations do not destabilize the PstB protein, we cloned these gene variants into the pRR48 plasmid and expressed them with a C-terminal 6X–His tag to be able to perform immunoblots. The plasmids were then introduced into BW26388, a strain that contains a Δ*pstB*::kan deletion mutation, and the strains were grown overnight in MOPS HiPi medium containing 50 μM IPTG and 50 μg/ml ampicillin and processed for Western blotting. As can be observed in Fig. 2b, the mutant proteins are at least as stable as the wild-type protein containing the 6X–His tag. We also confirm that the antibody is specific for the His-tag, as no band was observed when *pstB* was expressed without the added epitope.

Taken together, these observations are consistent with our model in that the conformation of PstB stabilized by the E179Q mutation signals a low-Pi environment by favoring an active kinase conformation in PhoR. The results with the Q160K mutant are consistent with a signaling state that maintains PhoR in its “phosphatase-on/kinase-off” conformation, which corresponds to growth in a high-Pi environment.

**Fig. 3:**
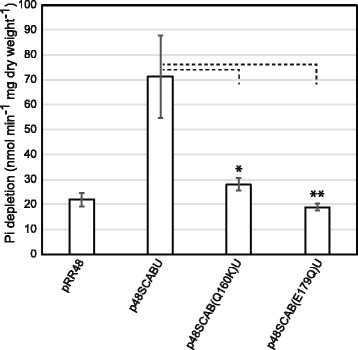
Pi uptake in the *pstB* mutant strains. Cultures of BW26337 cells with pRR48, p48SCABU, p48SCAB(Q160K)U or p48SCAB(E179Q)U were grown overnight in MOPS LoPi medium. After starving them of Pi and then adding K_2_HPO_4_, Pi uptake was measured. Each strain was assayed using two biological replicates in duplicate. Error bars represent ± the standard deviation. *, *P* < 0.05 between the p48SCABU and p48SCAB(Q160K)U strains and **, P < 0.01 between the p48SCABU and p48SCAB(E179Q)U strains, as determined by a two-tailed Student t test

Further support for the conformational signaling hypothesis comes by demonstrating that these transporters are nonfunctional, so Pi depletion assays were performed. Instead of measuring Pi uptake directly using ^32^Pi, we chose to measure Pi depletion from the medium using a nonradioactive method. Cells were grown in MOPS LoPi medium overnight and then incubated in Pi-free medium to exhaust the cells of intracellular Pi stores. Depletion assays were then started by adding Pi to a final concentration of 5.25 μM and then measuring the Pi content of filtered medium afterwards. As can be seen in Fig. 3, we compared the Pi depletion from the *pstB* mutants (Q160K and E179Q) to cells carrying the wild type gene and to cells carrying an empty vector. The strain containing the wild type gene showed a 3-fold increase of Pi depletion when compared to cells that have an empty vector. Moreover, both the mutants showed Pi depletion similar to that of the empty vector strain that was significantly different from the complemented strain. The background levels of Pi depletion are most likely due to transport through the constitutively expressed, low-affinity secondary transporters of *E. coli* called PitA and/or PitB*.* These results show that the Pst transporters containing either the Q160K or the E179Q mutation are nonfunctional. As the transporter containing the PstB(Q160K) protein is still capable of signaling a Pi-replete environment, even when Pi is limiting, we believe that these results are consistent with the proposal that signal transduction is independent of Pi transport and result from the predicted conformational changes in the transporter.

**Fig. 4:**
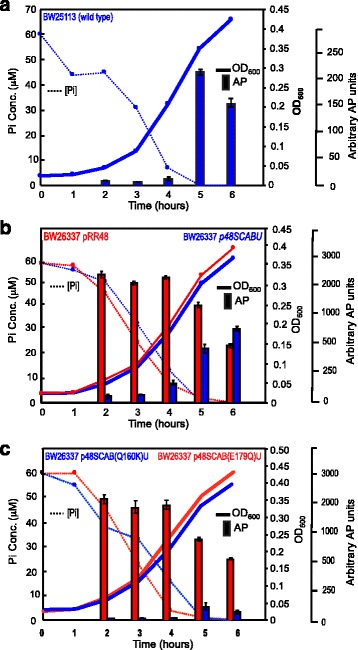
Induction of alkaline phosphatase expression upon Pi starvation. The Pi concentrations of the growth medium are represented by dashed lines, cell growth as measured by OD_600_ readings are shown with solid lines, and Pi-signaling, as measured by AP expression, is shown with solid vertical bars. The experiments reported in this figure were repeated three times on different days with similar results and we present the data from one trial. Measurements represent the averages of two technical replicates ± SD. **a** Wild type strain, BW25113 (**b**) BW26337 strain (Δ*pstSCABphoU*) carrying either pRR48 plasmid (Red) or p48SCABU (Blue). (**c**) BW26337 strain carrying either p48SCAB(Q160K)U (Blue) or p48SCAB(E179Q)U (Red)

We observed some differences in Pi-signaling from experiment to experiment, especially when growth times were extended in low-Pi medium, and wanted to know if those differences were maintained at different time points throughout the growth curve. Therefore, we followed Pi-signaling, as measured by AP expression, and measured medium Pi levels through the early stages of a growth curve for the wild type strain and the four experimental strains carrying plasmids. With this design, we were able to correlate Pi-signaling to environmental Pi levels in a single experiment. To perform the experiment, cells were grown in Pi-replete medium overnight and the following day, cells were washed in Pi-free medium, and then inoculated in defined medium containing 60 μM Pi, a concentration in which the Pho regulon is not induced. OD_600_ readings were taken at various time points thereafter and cells and spent medium were collected for AP analysis and for medium Pi concentrations, respectively. Figure 4a shows that in the wild-type cells, the AP expression turns on fully only when the environmental Pi levels were below ~5 μM (beginning from about 4.5 h of the growth curve). Figure 4b shows that in the Δ*pstSCABphoU* strain with an empty vector, the AP expression remains at a high level throughout this portion of the growth curve. The same strain carrying the p48SCABU plasmid showed low AP levels initially and induced AP expression when the Pi levels fell below ~10 μM (beginning from 4 h of growth curve). This is the same pattern as the wild-type strain; although the absolute AP values differ, probably because of the higher copy number of *pstSCAB-phoU* genes. Figure 4c shows that the PstB Q160K mutant always expressed low levels of AP, whereas the PstB E179Q mutant always expressed high levels of AP. We also noted a general decline in AP levels as the growth curve extended that were probably due to diminished synthetic potential. These results demonstrate that even when environmental Pi concentrations decrease to a level that normally activates the wild-type protein, a constant Pi-signal is transmitted by *pstB* mutants that are predicted to adopt stable conformations.

## Discussion

The Pho regulon has been studied for many decades, yet the molecular mechanisms by which *E. coli* cells recognize environmental Pi has remained unknown. Earlier work demonstrated that the signal for the activation of the Pho regulon is not a change in the intracellular concentration of Pi [32]. We have presented genetic evidence to support a model in which the signal corresponds to the alternate conformational states of the transporter. These PstSCAB conformations would normally be achieved as part of the Pi transport cycle, but in the present research, those conformations were achieved through mutagenesis of the *pstB* gene. We used bacterial AP activity as a reporter for the signaling state of the system. When the Δ*pstSCABphoU* strain was complemented with a medium-copy number plasmid harboring the *pstSCABphoU* genes we observed the same pattern of gene regulation as in the wild type strain, but the signaling levels were different. These differences were most likely due to differences in the copy numbers of the genes and to the loss of a genetic feedback loop that operates in the wild type strain. We also observed different signaling levels between the strains expressing the native version of *pstB* and the *pstB*Q160K and *pstB*E179Q variants. The strain expressing *pstB*Q160K displayed significantly higher AP levels than the native version grown in HiPi medium, but significantly less than the E179Q mutant. While the absolute AP values from the Q160K mutant were not the same as the strain expressing the native version of *pstB* in HiPi medium, the signaling pattern was consistent with a signaling state that inhibited full induction of the Pho regulon.

Recent work has demonstrated physical interactions between PstB, PhoU and PhoR [30, 31]. It has been suggested that a complex comprised of these proteins functions together to transmit information about environmental Pi levels to the transcriptional machinery through PhoB. We propose that PhoR senses the conformational states of the PstSCAB transporter within this complex to control its opposing kinase and phosphatase activities (See Fig. 1a). The inward-facing, open conformation of the transporter would therefore interact with PhoR to promote its phosphatase activity. The outward-facing, closed conformation of the transporter would be predicted to interact with PhoR to promote its kinase activity.

We attempted purification of the PstSCAB proteins for biochemical assays to provide further evidence in support of our hypothesis, but were largely unsuccessful. Importantly, introducing His-tags on either the N- or the C-terminus of PstB altered signal transduction activity (data not shown; Kristi Johns Master’s Thesis, Brigham Young University). However, equivalent mutations to those we employed here for PstB have been used in numerous structural and biochemical studies on other ABC transporters. For example, when MalK Q140K was incorporated into a transport complex with MalF and MalG, it showed very low ATPase activity and was completely defective in maltose transport [37, 48]. It was suggested that this variant of MalK locked the full transporter into a ground state. In separate studies on GlcV and MJ0796, which are the isolated NBDs from the glucose transporter from *Sulfolobus sulfataricus* and a transporter of unknown cargo from *Methanococcus jannashii,* the conserved glutamate residues in the Walker B box were changed to glutamines (equivalent to the E179Q mutation in PstB) [49, 50]. In both cases, these mutations were shown to stabilize NBD dimerization, which corresponds to the closed conformation of PstB. A similar Glu to Gln mutation was introduced into the MsbA lipid flippase from *Salmonella typhimurium,* which was then reconstituted into nanodiscs [51]. When examined by luminescence resonance energy transfer, this full transporter was also shown to be in a closed, outward-facing conformation. While there is great diversity in the many ABC transporters found in nature, it is reasonable to propose that the mutations that were introduced into *pstB* may indeed have the proposed effects on the conformation of the PstSCAB transporter.

## Conclusions

The PstSCAB transporter not only imports Pi into the cell, but it also signals information concerning environmental Pi to the transcription machinery. PstSCAB adopts multiple conformations as part of itsnormal transport cycle. These alternate conformations are then sensed by PhoR in a mechanism that requires the PhoU protein in a signaling comlex. This sensory mechanism thus controls the alternate autokinase and phospho-PhoB phosphatase activities of PhoR, which ultimately control the signaling state of the response regulator PhoB.

## Abbreviation

Pi: Phosphate
